# The Relationships Between Cognitive Reserve and Creativity. A Study on American Aging Population

**DOI:** 10.3389/fpsyg.2018.00764

**Published:** 2018-05-23

**Authors:** Barbara Colombo, Alessandro Antonietti, Brendan Daneau

**Affiliations:** ^1^Neuroscience Lab, Champlain College, Burlington, VT, United States; ^2^Department of Psychology, Catholic University of the Sacred Heart, Milan, Italy

**Keywords:** cognitive reserve, aging, creativity, WAIS, flexibility

## Abstract

The Cognitive Reserve (CR) hypothesis suggests that the brain actively attempts to cope with neural damages by using pre-existing cognitive processing approaches or by enlisting compensatory approaches. This would allow an individual with high CR to better cope with aging than an individual with lower CR. Many of the proxies used to assess CR indirectly refer to the flexibility of thought. The present paper aims at directly exploring the relationships between CR and creativity, a skill that includes flexible thinking. We tested a sample of 72 adults (aged between 45 and 78) assessing both their level of CR and their creativity. To evaluate CR we used the proxies commonly used in literature, namely, three subtests from the WAIS (vocabulary, similarities, and digit span) and the years of education. We also used an *ad-hoc* test asking people to report how frequently they tend to perform activities that are believed to increase CR. We used verbal creativity tasks (alternative uses and generation of acronyms) to assess individual levels of creativity. We asked participants to describe their main occupation (present or past) and coded each occupation as creative or not creative. Results (controlling for age-related differences) showed that scores from the WAIS correlated positively with creativity performance, even though correlations varied across the subtests. Focusing on the frequency and type of activities that people perform, and comparing individuals who have or had a creative job to those with a routine job, a clear relationship between creativity and CR emerged. This effect was more relevant than the level of job complexity. Implications for the study of CR and aging are discussed.

## Introduction

The reserve hypothesis has been introduced by Stern (2002, 2006, 2009) to explain individual differences that allow some people to cope better than others with brain damage. This line of research has been inspired by the evidence that in some elderly, despite the presence of considerable brain pathology, no clinically-observable signs or symptoms of a disease are reported (Mortimer et al., 2003). The reserve model explains this disparity by referring to differences in the cognitive processes or neural networks underlying task performance. People with higher reserve can “optimize or maximize performance through differential recruitment of brain networks, which perhaps reflect the use of alternate cognitive strategies” (Stern, 2002, p. 451). Interestingly, Stern (2009) also noted that the reserve is relevant not just to the onset of dementia or other neurological, age-related diseases, but also to normal aging, as it allows the aging population to cope more efficiently with age-related brain changes.

The reserve hypothesis refers to two different models (see Stern, 2002, 2006, 2009 for extensive reviews): the passive and the active one. The passive model is also defined as the “Brain Reserve” and refers to the positive relationship between the brain size and the ability to cope with pathology without showing signs of clinical impairment (Stern, 2009). The active model is usually referred to as “Cognitive Reserve” (CR) and suggests that different life experiences (such as education, occupation, and cognitively-stimulating leisure activities) provide a shield against the effects of brain damage or pathology, helping the individual to cope by enlisting compensatory processes and slowing down memory decline in normal aging (Stern, 2009).

In this paper we are going to refer to the active model, focusing on CR and its possible relationship with creativity. This relationship can be speculatively inferred by reflecting on the proxies that are commonly used to measure CR. The reason why proxies are needed is that CR cannot be directly measured, as is the case for the brain reserve. For this reason, it is commonly assessed indirectly by evaluating experiences and activities that are believed to increase it. As mentioned above, the most commonly-used proxy measures refer to educational level and literacy (Stern et al., 1992; Manly et al., 2003, 2005), occupational status, with a specific attention to occupational complexity (Stern et al., 1994; Richards and Sacker, 2003; Staff et al., 2004), and engagement in cognitively-stimulating leisure activities (e.g., Wilson et al., 1999; Aartsen et al., 2002; Mousavi-Nasab et al., 2014; Colombo et al., 2018). The cohesion of social networks (Fratiglioni et al., 2000; Bennett, 2006; Colombo et al., 2018) and personality variables have been incorporated into CR, too (Bennett et al., 2006; Wilson et al., 2007). A good example of how these proxies can be integrated together can be found in the critical evaluation of the Cognitive Reserve Index questionnaire developed and validated by Nucci et al. (2012).

A recent meta-analysis (Opdebeeck et al., 2016) supports the idea that indices of CR are related to cognitive function in some different domains, although the reported associations are modest. The authors stressed the need for further studies to more comprehensively investigate the relationships between CR and specific, well-defined, cognitive functions in healthy and clinical populations. Creativity might be one of these functions.

Starting from these remarks, we decided to focus on the possible relationship between CR (assessed by using education levels, occupation complexity, and number and frequency of leisure activities, as well-intelligence tests) and creativity (assessed using tasks asking to list as many responses as possible to given stimuli, as often occurs in the assessment of creative skills) in a healthy aging population. The reason for exploring this relationship originates from a definition of creativity that, trying to go beyond differences among different theoretical perspectives, highlights the common cognitive principles behind all of them (Antonietti and Colombo, 2013, 2016). From this perspective, creativity can be declined as three mental operations: widening (the tendency to keep an open mind and be able to deal with a high number of elements), connecting (the capacity to establish relationships among different elements and to combine them in unusual ways), and reorganizing (being able to change perspective and invert relationships among elements). CR, as discussed above, has been defined as a factor that allows the aging population to use alternative strategies to better cope with age-related brain damages. Skills required for doing so appear to be similar to the ones, listed above, that characterize the creative process. Accessing and applying alternative strategies require an individual to be able to keep an open mind, establish new and unusual relationships, and change perspective as requested.

We were hence hypothesizing to find a positive relationship between levels of CR and levels of creativity in our participants. A recent study (Palmiero et al., 2016) investigated the possibility of using creativity as a proxy for CR. Results highlighted that verbal creativity, but not visual creativity, predicts CR. Other recent studies explored more indirectly constructs that could be related to creativity. If is true that creative thinking can be seen as the result of the concurrent activation of several neural networks (see, for example, Beaty et al., 2015), the dorsolateral prefrontal cortex (DLPFC) in particular appears to regulate some aspects of the creative process (e.g., Chrysikou et al., 2013; Iannello et al., 2014; Colombo et al., 2015; Weinberger et al., 2017) and flexibility in thinking (Oldrati et al., 2016, 2018). For this reason, studies reporting a role of the DLPFC in moderating CR are particularly relevant to our investigation. Roldán-Tapia et al. (2012) and Arcara et al. (2017) focused on the relationships between the CR and several cognitive and executive functions. Results highlighted that CR levels (mainly education in Arcara and colleagues' study) contribute significantly to the performance in tasks that refer back to functions mainly related to the dorsolateral prefrontal area. Another study applying rTMS over the DLPFC (Manenti et al., 2011) on a sample of healthy older adults led the authors to conclude that left DLPFC rTMS during encoding only resulted in a disruptive effect among elders exhibiting low memory performance but not among high performing elders, suggesting that the underlying mechanisms in the latter group imply a more distributed recruitment of the contralateral DLPFC to counteract age-related functional brain loss.

Studies exploring, directly or indirectly, the relationship between CR and creativity report are at an exploratory level, hence collecting more data using different measures, as suggested by Palmiero et al. (2016), to confirm these initial findings seems to be relevant.

## Methods

### Sample

Seventy-two healthy individuals, aged between 42 and 78 (Mean = 58.67; *SD* = 12.31), joined the study. They were not balanced by gender (women = 66.7%). Participants were recruited through posts on local newspapers and by contacting local senior centers. All participants were from North and central Vermont and had a high or middle SES. We followed the APA suggested best practices for measuring socioeconomic status (http://www.apa.org/pi/ses/resources/class/measuring-status.aspx). We measured Education and Occupation (used to assess the CR as well, see details below) and also asked participants to report the average family income. Participants who reported symptoms or diagnosis linked to dementia or other age-related disorders were excluded.

As a compensation for the time participants spent in taking part to the present study, after the assessment they received a free 15-week program designed to enhance the CR by the way of suggesting relevant activities targeting different proxies reported in the literature as effective to increase CR.

### Tools

#### Wechsler adult intelligence scale (WAIS)

Subtests from the WAIS-IV (Wechsler, 2008)—namely, Vocabulary, Similarities, and Digit Span (forward and backward)—have been used. The Vocabulary subset assesses word knowledge and verbal concept formation. The Similarities subtest measures verbal concept formation and reasoning. The Digit Span subtest focuses on working memory. These subtests have been reported and used in the literature as a measure of CR (see, for example, Corral et al., 2006; Solé-Padullés et al., 2009; Roldán-Tapia et al., 2012).

The assessment was performed following the instructions indicated in the manual of the WAIS-IV. The conditions of test application were constant for all participants. Subtests were applied individually in one session. A trained research assistant performed all the evaluations.

#### CoRe-T

To include the specific proxies linked to CR and to assess creative thinking, we used an *ad-hoc* questionnaire, CoRe-T (Cognitive Reserve Test). The questionnaire includes two main sections (self report and creative tasks) and five subsections:

1. Self-report data
  - Education: We asked people to report the years of completed education, including vocational training. Participants were also asked to list each degree, diploma, and certificate together with the year it was earned.
  - Leisure activities: Participants were presented with a list of 17 leisure activities derived from the ones reported in the literature as linked to CR (see Appendix 1 for the complete list of activities). For each activity, they were asked to rate (on a 5-point Likert scale, where 1 corresponded to rarely/never and 5 to often/every day) the frequency of performing that activity. Respondents were also asked to report an estimate of the numbers of years they have been performing that specific activity. Years of activity were defined as the highest consecutive number of years performing the activity by using a 3-point Likert scale (1 = 1 year or less; 2 = 2–5 years; 3 = 5 years or more). A total score of CR as represented by the frequency of performing leisure activities was devised by computing the mean score of the reported frequencies for all 17 leisure activities listed in the CoRe-T.
  - Occupation history: Participants were asked to list the general type of occupation, the specific position(s) they had, and the number of years they have been working in each position.
2. Creative tasks Two tasks commonly used to assess verbal creative abilities were chosen to be included in the CoRe-T.
  - Acronyms (Guilford, 1967): Participants were given 5 min to list all the terms that can fit into the three given acronyms (SOS—OMG—TGIF). The terms had to make sense together.
  - Alternative uses (Guilford, 1967; Torrance, 1990): Participants were given 5 min to list as many different, interesting or unusual usages for an empty plastic bottle as they could.

Participants' answers to the two creative tasks have been scored following the guidelines derived from the Torrance Test of Creative Thinking (Torrance, 1990). For each task we computed a fluidity score by counting the numbers of valid answers. Invalid answers for the acronym task were defined as answers using non-existing words or using terms that did not make sense together. Invalid answers for the alternative use tasks were answers according to which bottles are used as bottles (hence not providing any new use) or where the use of bottles is missing. Two researchers coded all the answers independently. An originality scored was computed as well. After reading all the answers for each task in order to derive a list of the most common answers, a list of original (i.e., not listed among the most frequently reported uses) answers has been compiled by two researchers. Each individual answer was then coded as original (1) or not original (0). A final score was computed by adding the number of original answers. Cases of disagreement in scoring responses to the creative tasks were discussed and resolved case by case by the two researchers.

#### Procedure

The study has been approved by Champlain College IRB committee.

Participants who expressed an interested in being involved in the study were contacted by a member of the research team to check for eligibility criteria. Eligible participants booked an appointment to be individually tested. Before starting the assessment, participants were given the Informed Consent and were asked to sign it. They were also given the possibility to ask any questions they might have.

After this preliminary phase, the three subtests of the WAIS-IV were administered, followed by the CoRe-T. At the end of the session, participants were offered to have either a printed or a digital copy of a training program designed to increase their CR. The program suggests weekly activities targeting different proxies reported in the literature as effective to increase CR.

## Results

Before running specific analyses, we checked the dataset looking for possible outliers. None emerged, so we proceeded to the analyses keeping all the participants included in the original sample.

### Effects of CR on creativity

We performed a series of linear regressions, using CR proxies (i.e., WAIS-IV subtests scores, years of completed education, and frequency of involvement in leisure activities) as predictors and the scores derived from the creative tasks as dependent variables. Least squared regression was weighted by age. Overall CR proxies were able to predict performance in the creative tasks, but different proxies explained different aspects of the performance (see Table 1).

**Table 1:** Liner Regression Model considering the effects of CR proxies on creative performance.

		***b***	***SEb***	**β**
**ACRONYM FLUIDITY**				
	Constant	−15.30	2.77	
	Similarities	0.49	0.08	0.53^***^
	Digital span 1	0.26	0.21	0.09
	Digital span 2	−0.30	0.17	−0.12
	Vocabulary	0.13	0.07	0.22
	Years of education	0.05	0.15	0.03
	Frequency of leisure Activities	3.76	0.50	0.45^***^
*R*^2^ = 0.78; *p* < 0.001; ^***^p < 0.001				
**ACRONYM ORIGINALITY**				
	Constant	−18.35	2.11	
	Similarities	0.45	0.06	0.49^***^
	Digital span 1	0.45	0.16	0.15^**^
	Digital span 2	−0.45	0.13	−0.18^**^
	Vocabulary	0.21	0.06	0.36^***^
	Years of education	0.00	0.11	0.00
	Frequency of leisure Activities	3.55	0.38	0.43^***^
*R*^2^ = 0.87; *p* < 0.001; **p < 0.01; ^***^p < 0.001				
**ALTERNATIVE USE—FLUIDITY**				
	Constant	−2.37	2.67	
	Similarities	0.15	0.08	0.17
	Digital span 1	−0.98	0.20	−0.34^***^
	Digital span 2	0.23	0.16	0.10
	Vocabulary	0.25	0.07	0.44^**^
	Years of education	0.48	0.14	0.29^**^
	Frequency of leisure Activities	1.09	0.49	0.14^*^
*R*^2^ = 0.77; *p* < 0.001; ^*^p < 0.05; ^**^p < 0.01; ^***^p < 0.001				
**ALTERNATIVE USE—ORIGINALITY**				
	Constant	−5.59	2.98	
	Similarities	0.37	0.09	0.45^***^
	Digital span 1	−0.46	0.22	−0.73^*^
	Digital span 2	0.01	0.18	0.01
	Vocabulary	0.12	0.08	0.22
	Years of education	0.29	0.16	0.19
	Frequency of leisure Activities	1.40	0.55	0.19^**^
*R*^2^ = 0.67; *p* < 0.001; ^*^p < 0.05; ^**^p < 0.01; ^***^p < 0.001				

To investigate the possible specific effects of different types of leisure activities, we computed frequencies scores of different categories of leisure activities: creative (i.e., playing music, making art, attending arts events, etc.), cognitive (i.e., playing crosswords, using technology to look up information, taking care of family budget, etc.), physical (i.e., exercising, gardening, practicing a sport, etc.), and social (i.e., being part of a club, taking care of a family members, attending social events, etc.).

Then we run the same regression analysis, using the categories of leisure activities as predictors and the scores of the cognitive tasks as dependent variables. Least squared regression was weighted by age. Results are reported in Table 2.

**Table 2:** Liner Regression Model considering the effects of different categories of leisure activities on creative performance.

		***b***	***SEb***	**β**
**ACRONYM FLUIDITY**				
	Constant	−3.29	2.32	
	Creative activities	3.72	1.05	0.60^***^
	Cognitive activities	0.42	0.80	0.05
	Physical activities	−0.49	0.61	−0.08
	Social activities	0.59	0.80	0.11
*R*^2^ = 0.47; *p* < 0.001; ^***^p < 0.001				
**ACRONYM ORIGINALITY**				
	Constant	−2.84	2.28	
	Creative activities	3.72	1.05	0.60^***^
	Cognitive activities	0.42	0.80	0.05
	Physical activities	−0.49	0.61	−0.08
	Social activities	0.59	0.80	0.11
*R*^2^ = 0.47; *p* < 0.001; ^***^p < 0.001				
**ALTERNATIVE USE—FLUIDITY**				
	Constant	6.53	2.69	
	Creative activities	2.48	1.22	0.42^*^
	Cognitive activities	−1.96	0.93	−0.26^*^
	Physical activities	1.61	0.71	0.28^*^
	Social activities	−0.34	0.92	−0.07
*R*^2^ = 0.23; *p* < 0.01; *p < 0.05				
**ALTERNATIVE USE—ORIGINALITY**				
	Constant	3.05	2.57	
	Creative activities	0.74	1.17	0.14
	Cognitive activities	−0.78	0.89	−0.11
	Physical activities	1.60	0.68	0.30^*^
	Social activities	0.61	0.87	0.13
*R*^2^ = 0.18; *p* < 0.01; ^*^p < 0.05				

When examining the creative performance in response to the acronym task, practicing creative activities improved the task performance, positively affecting both fluency and originality, where no type of other leisure activities apparently influenced creativity. The alternative tasks task returned a different picture. Fluidity was positively affected by performing creative tasks and by being physically active and was negatively affected by being engaged in cognitive activities. Originality was positively affected by being physically active.

### Effects of creative activities on CR

We also investigated the possible effects of activating creativity throughout the life on the different proxies of CR. Participants' responses to section 3 (Occupation history) of the CoRe-T were analyzed and different occupations were coded as creative or non-creative. Jobs were coded as creative if they were not routine tasks, required constant flexibility of thoughts, changes of perspective, and creation of new and innovative ideas/solutions. Cases of disagreement were discussed and resolved case by case by the two researchers who were in charge of all scoring procedures. Examples of creative jobs were: manager of an art gallery, musician, college professor, and high school teacher (these last examples have been discussed but ultimately defined as creative because of the necessity of constantly vary course contents or class activities and adopt different perspectives when interacting with different students). Examples of non-creative jobs were: lab technician, post office employer or director, bank teller. An overall evaluation (creative job vs. non-creative job) was computed for each participant, considering the prevalent type of occupations and the numbers of years that each occupation was covered by the participant.

We performed a MANOVA considering CR proxies as dependent variables, the main type of occupation (creative vs. non-creative) as a fixed factor, and age as a covariate. Means scores and standard deviations are reported in Table 3.

**Table 3:** Mean scores and standard deviation for the different cr proxies according to occupation creativity level.

**CR Proxy**	**Occupation creativity level**	**Mean**	**Std. Deviation**
Similarities	Non-creative	14.56	5.75
	Creative	21.56	3.06
Digit Span Direct	Non-creative	10.33	2.39
	Creative	10.67	1.07
Digit Span Reverse	Non-creative	7.00	2.02
	Creative	8.56	2.14
Vocabulary	Non-creative	30.22	8.11
	Creative	41.33	6.20
CR on the base of frequency of LA	Non-creative	2.67	0.61
	Creative	3.11	0.60

Having been employed in creative jobs appears to have a direct effect on several proxies of CR. The factors more affected were Similarities [*F*_(1, 69)_ = 35.40; *p* < 0.001; η^2^ = 0.34] and Vocabulary [*F*_(1, 69)_ = 35.69; *p* < 0.001; η^2^ = 0.34], followed by Reverse Digit Span [*F*_(1, 69)_ = 9.12; *p* < 0.01; η^2^ = 0.12] and frequency of leisure activities [*F*_(1, 69)_ = 7.14; *p* < 0.01; η^2^ = 0.09]. Performance on the Direct Digit Span task was the only proxy not affected by the occupation type.

Since the complexity of occupation, as discussed in the Introduction, has been reported as a factor influencing CR, we also run a more fine analysis, considering the level of complexity of occupation as well as the creative components of it. To do so, we categorized the occupations according to level of complexity by referring to the description of the specific position each individual reported in section 3 of the CoRe-T. We divided jobs into 4 categories: low complexity and not creative (e.g., post office employer); low complexity and creative (e.g., nanny); high complexity and not creative (e.g., director of a car rental agency); high complexity and creative (e.g., director of a music series). We run another ANOVA, considering CR proxies as dependent variables, the main type of occupation (divided into the four levels described above) as a fixed factor, and age as a covariate. Means scores and standard deviations are reported in Table 4.

**Table 4:** Mean scores and standard deviation for the different CR proxies according to occupation creativity and complexity level.

**CR Proxy**	**Occupation creativity and complexity levels**	**Mean**	**Std. Deviation**
Similarities	Low complexity and not creative	13.80	6.20
	Low complexity and creative	25.00	1.07
	High complexity and not creative	15.50	5.19
	High complexity and creative	20.57	2.71
Digit span direct	Low complexity and not creative	9.80	2.28
	Low complexity and creative	10.00	0.00
	High complexity and not creative	11.00	2.42
	High complexity and creative	10.86	1.14
Digit span reverse	Low complexity and not creative	7.60	1.23
	Low complexity and creative	7.00	1.07
	High complexity and not creative	6.25	2.57
	High complexity and creative	9.00	2.18
Vocabulary	Low complexity and not creative	29.00	6.99
	Low complexity and creative	38.50	4.81
	High complexity and not creative	31.75	9.33
	High complexity and creative	42.14	6.38
Cognitive reserve on the base of frequency of LA	Low complexity and not creative	3.02	0.46
	Low complexity and creative	3.62	0.34
	High complexity and not creative	2.23	0.49
	High complexity and creative	2.97	0.58

The between-subject test highlighted similar main effects as the ones emerged from the first ANOVA. They type of job influenced the performance on Similarities test [*F*_(3, 67)_ = 15.86; *p* < 0.001; η^2^ = 0.42], Reverse Digit Span [*F*_(3, 67)_ = 6.95; *p* < 0.001; η^2^ = 0.24], Vocabulary [*F*_(3, 67)_ = 13.50; *p* < 0.001; η^2^ = 0.36], and Frequency of Leisure Activities [*F*_(3, 67)_ = 13.28; *p* < 0.001; η^2^ = 0.37]. No effect of the performance of the Direct Digit Span emerged [*F*_(3, 67)_ = 1.97; *p* = 0.13; η^2^ = 0.08].

A pairwise comparison, performed using Bonferroni correction, showed significant differences among the levels of the independent variable. Mean differences and standard errors are reported in Table 5. Creativity levels rather than complexity were reliable predictors of the levels of CR proxies. Individuals with creative occupation performed better in the Similarities and Vocabularies tests than individuals who had non-creative occupations, and this was true regardless of the complexity of the job. The same was true for the frequency of leisure activities: Participants who had creative jobs reported to be involved more frequently in different types of leisure activities. No significant difference emerged for the Direct Digit Span test. Only creative jobs characterized by high complexity allowed individuals to score better in the Reverse Digit Span test.

**Table 5:** Pairwise comparison; mean difference and se for the different CR proxies according to occupation creativity and complexity level.

			**Mean Difference**	**Std. Error**	***p***
Similarities	Low complexity and not creative	Low complexity and creative	−11.03	1.83	<0.001
		High complexity and not creative	−2.12	1.49	0.95
		High complexity and creative	−6.47	1.29	<0.001
	Low complexity and creative	Low complexity and not creative	11.03	1.83	<0.001
		High complexity and not creative	8.91	1.93	<0.001
		High complexity and creative	4.56	1.75	0.07
	High complexity and Not creative	Low complexity and not Creative	2.12	1.49	0.95
		Low complexity and creative	−8.91	1.93	<0.001
		High complexity and creative	−4.35	1.43	0.02
	High complexity and creative	Low complexity and not creative	6.47	1.29	<0.001
		Low complexity and creative	−4.56	1.76	0.07
		High complexity and not creative	4.35	1.43	0.02
Digit Span	Low complexity and not creative	Low complexity and creative	−0.18	0.76	1.00
		High complexity and not creative	−1.24	0.620	0.29
		High complexity and creative	−1.03	0.54	0.36
	Low complexity and creative	Low complexity and not creative	0.18	0.76	1.00
		High complexity and not creative	−1.06	0.80	1.00
		High complexity and creative	−0.844	0.730	1.00
	High complexity and not creative	Low complexity and not creative	1.24	0.62	0.29
		Low complexity and creative	1.06	0.80	1.00
		High complexity and creative	0.22	0.59	1.00
	High complexity and creative	Low complexity and not creative	1.03	0.54	0.36
		Low complexity and creative	0.84	0.73	1.00
		High complexity and not creative	−0.22	0.59	1.00
Digit Span Reverse	Low complexity and not creative	Low complexity and creative	0.58	0.83	1.00
		High complexity and not creative	1.39	0.67	0.26
		High complexity and creative	−1.43	0.59	0.10
	Low complexity and creative	Low complexity and not creative	−0.5859	0.8319	1.00
		High complexity and not creative	0.8029	0.8729	1.00
		High complexity and creative	−2.01	0.80	0.08
	High complexity and not creative	Low complexity and not creative	−1.39	0.67	0.26
		Low complexity and creative	−0.80	0.87	1.00
		High complexity and creative	−2.81	0.65	<0.001
	High complexity and creative	Low complexity and not creative	1.43	0.59	0.10
		Low complexity and creative	2.01	0.80	0.08
		High complexity and not creative	2.81	0.65	<0.001
Vocabulary	Low complexity and not creative	Low complexity and creative	−9.14	2.91	0.01
		High complexity and not creative	−3.64	2.36	0.77
		High complexity and creative	−12.51	2.05	<0.001
	Low complexity and creative	Low complexity and not creative	9.14	2.91	0.01
		High complexity and not creative	5.50	3.06	0.45
		High complexity and creative	−3.37	2.79	1.00
	High complexity and not creative	Low complexity and not creative	3.64	2.36	0.77
		Low complexity and creative	−5.50	3.06	0.46
		High complexity and creative	−8.87	2.28	0.01
	High complexity and creative	low complexity and not creative	12.51	2.05	<0.001
		low complexity and creative	3.36	2.79	1.00
		high complexity and not creative	8.87	2.28	0.01
Cognitive Reserve on the base of frequency of LA	Low complexity and not creative	Low complexity and creative	−0.58	0.21	0.05
		High complexity and not creative	0.76	0.17	<0.001
		High complexity and creative	0.07	0.15	1.00
	Low complexity and creative	Low complexity and not creative	0.58	0.21	0.05
		High complexity and not creative	1.35	0.22	<0.001
		High complexity and creative	0.66	0.20	0.01
	High complexity and not creative	Low complexity and not creative	−0.76	0.17	<0.001
		Low complexity and creative	−1.35	0.22	<0.001
		High complexity and creative	−0.69	0.17	0.01
	High complexity and creative	Low complexity and not creative	−0.07	0.15	1.00
		Low complexity and creative	−0.66	0.20	0.01
		High complexity and not creative	0.69	0.17	0.01

## Discussion and conclusions

The present study aimed at exploring the relationships between CR and creativity. Literature about the role of different proxies used to assess CR highlighted that most of them share the common characteristic of allowing the aging population to use alternative strategies of thought. This would help the elder to better cope with age-related brain injuries. Skills required for using these alternative strategies appear to be similar to the ones that characterize the creative process. To be more precise, accessing and applying alternative strategies require an individual to be able to keep an open mind, to establish new and unusual relationships, and to change the perspective when required. These mental operations have been used to define a comprehensive creative process (Antonietti and Colombo, 2013, 2016).

Starting from these similarities, we were expecting to find a positive relationship between CR and creativity. We explored both a direct and reverse relationship, and in both cases our hypothesis was confirmed.

We started by exploring the effects of the proxies most commonly used in literature to assess CR and explore their possible influence on the performance of creative tasks (verbal creativity). Results highlighted that the proxies influenced performance of creative tasks but in different ways. Individual cognitive abilities, as measured by the WAIS subtests, had significant effects on specific aspects of the creative tasks, depending on the ability that was required the most to provide good answers. For example, higher scores in the Vocabulary subtest led participants to be more original when inventing new synonyms and produce more alternative uses of empty water bottles. This result is coherent with the definition of CR as the capacity to recruit different networks, optimizing the performance, and reflecting the use of alternate cognitive strategies as needed by the task at hand (Roldán-Tapia et al., 2012). This finding also confirms the results by Palmiero et al. (2016), who found that verbal creativity (the same that we assessed using the CoRe-T) specifically predicts the level of CR. Interestingly, the performance in the Digit Span test showed often a negative relationship with the creative performance. It is likely that individuals were not relying so much on the use of working memory while facing creative tasks. This result is coherent to some extent with the results from Arcara et al. (2017), who reported that a general CR index did not predict abstract math ability. Even if the Digit Span test is not a math test *per se*, it requires the activation of neural networks related to mathematical thinking, together with working memory (Raghubar et al., 2010). It is worth noticing that literature showed a positive relationship between working memory and creative performance (e.g., De Dreu et al., 2008, 2012; Takeuchi et al., 2011), but it looks like our participants preferred relying on processes more closely associated with long-term memory (i.e., their vocabulary skills). Since most of the studies reported in literature used a younger population as a sample, this could be an interested age-related effect to be further investigated in future studies.

The frequency of leisure activities was the proxy that had always a significant positive effect on the creative performance. This could have been because diversifying everyday leisure activities helps people to generate many different ideas and to change the mental perspective frequently. To support this possible reading of the finding reported above, we investigated the specific role of different categories of leisure activities to test if we could find a specific effect due to different types of leisure activities people were involved in. Results highlighted that, while (as it could be expected) it was the frequency of engaging in creative activities that predicts best the performance in the acronym task, the performance of the alternative uses task was significantly influenced by the frequency of several types of creative activities. Fluency was significantly predicted by all categories other than by social activities. Practicing cognitive actives showed a negative but significant relationship with the number of alternative uses suggested by participants. Someone might argue that there is something about engaging in creative activities that make individuals slightly more conservative in their choice of responses to divergent thinking tasks. Being more involved in creative activities should elicit the opposite behavior since individuals should learn that being free to generate ideas can be useful and productive, and hence face tasks like the Alternative Uses with this same perspective. Following this line of thoughts, it is quite interesting to remark that the originality of uses was positively related to the frequency of practicing physical activity. Being involved in a variety of leisure activities might induce people to figure out alternative uses for objects since these individuals are probably exposed to more and more differentiated situations. The specific link between practicing physical activities and being more original in alternative uses task can be partially explained by referring to existing literature reporting a direct positive relationship between practicing physical activities and increasing creative thinking, at least in children (for a review see Best, 2010; for a meta-analysis see Fedewa and Ahn, 2011). Future studies should explore better this relationship in an older population. The only review on the topic (Angevaren et al., 2008) explores mainly the effect of cardiovascular activities on cognitive function in the elderly population. From our data, it is clear that most of the spontaneous physical activities reported by our participants were not cardiovascular, but more moderate, if constant. The fact that we found a specific strong effect on the originality of answers in our sample could also be explained by observing that gardening was one of the activities included in this category of leisure activities. Empty plastic bottles could be used in many different ways while gardening. Specific gardens or project might lead to inventing different uses according to specific needs, and this might help to explain this result. Exploring this effect using a different version of the alternative uses tasks could help clarifying this point.

We also explored the possible effect of being involved in a creative job over time on CR. Again, we were expecting a positive effect, given the overlapping between the cognitive functions involved in cognitive processes and the ones that promote CR. The type of job (creative vs. non-creative) influenced almost all the proxies of CR (both cognitive, as assessed by the WAIS subtests, and behavioral, as reflected by the frequency of being involved in leisure activities). The most interesting finding emerged when comparing creative and non-creative job while taking into consideration the complexity of the position. This more refined analysis was performed on the basis of literature findings suggesting that job complexity can influence CR (Stern et al., 1994; Richards and Sacker, 2003; Staff et al., 2004). Our findings showed that the creative component, more than the complexity *per se*, affects CR. This result has a direct link with our main research question, which was focused on the similarities between the mechanisms that have been hypothesized to be at the basis of CR and to promote it and the ones that have been supposed to be the common mental functions underlying creative thinking.

The study presents some limitations that could be addressed by future research. First of all our sample was recruited only from one State, which is characterized by a very rural environment and has a high percentage of active elders. This might have affected the results. More data from different environments should be collected. A second limitation is the size of our sample. Even if the results always reached acceptable power levels, the sample *per se* was quite small. Our results should be replicated with a larger sample. The current results should be seen as a first promising step. Our participants also had high or medium SES: Considering a population from a low SES could promote a better understanding of CR and could help reaching conclusions that could be generalized. We also decided not use standardized creativity tests, but adopt alternative versions of creative tasks. Even if this choice can be read as a limitation, using alternative versions of standard tasks is a relatively common practice (Guilford, 1967; Torrance, 1990; Colombo et al., 2015). We decided to pick this route, which allows us to circumvent copyright issues, because we aim at having the CoRe-T available to use as a standalone test for CR that includes creative tasks.

This study, though preliminary mainly be because of the limited sample size, highlighted some new aspects that can help clarify the specific nature of the cognitive mechanism underlying CR, especially its close relationship with creative thinking and the level of creativity of previous occupations, a link that has not been explored so far, to our knowledge. This might be useful in devising program to increase CR by focusing on creative tasks, which could be perceived as engaging and motivating for the elderly population.

Our data also offer suggestions on an alternative way to assess CR. Creative tasks could be added as proxies to assess CR. This has been already suggested in literature (see Palmiero et al., 2016) but our data allow to be even more detailed in suggesting how to include creativity in a CR assessment. A combination of creative tasks and a focus on lists of leisure activities that could be easily categorized to examine the frequency of creative vs. cognitive, vs. physical, vs. social activities could be beneficial both in clinical and experimental settings. More data from an instrument like the CoRe-T, used in the current study, could help researchers moving in this direction.

## Ethics statement

This study was carried out in accordance with the recommendations of APA ethical guidelines with written informed consent from all subjects. All subjects gave written informed consent in accordance with the Declaration of Helsinki. The protocol was approved by the Champlain College IRB Committee.

## Author contributions

BC designed the research, helped with data collection and coding, analyzed the data, wrote the paper. AA helped to design the research, discussed the findings, revised the paper. BD helped designing data collection, collecting, and coding the data.

### Conflict of interest statement

The authors declare that the research was conducted in the absence of any commercial or financial relationships that could be construed as a potential conflict of interest.

## Appendix 1—list of leisure activities assessed using the CoRe-T

Frequency of activity is based on an average week.

**Table:**

1	2	3	4	5
Rarely/Never				Often/Everyday
Activity			Frequency	Years

**Table:**

**Example: Playing Chess**	**4**	**2**
Reading (Magazines, Newspapers)		
Use of Technology (Cellphones, Computers)		
Use of Other Language		
Physical Exercise		
Bank Account Management		
Puzzles (Crossword, Word Search)		
Reading (Books, Novels)		
Music (Listening, Playing)		
Gardening		
Cooking		
Art (For self)		
Art (For Showcase)		
Social Based Clubs (Book Clubs, Knitting Club, Etc.)		
Taking Care of Others		
Taking Care of Pets		
Managing Family Budget		
Playing Sports		
Other:		
